# The diagnostic value of intravenous contrast computed tomography in addition to plain computed tomography in dogs with head trauma

**DOI:** 10.1186/s12917-021-02764-6

**Published:** 2021-01-22

**Authors:** Yasamin Vali, Ingrid Gielen, Sarang Soroori, Eberhard Ludewig

**Affiliations:** 1 Diagnostic Imaging, Department for Companion Animals and Horses, University of Veterinary Medicine Vienna (Vetmeduni), Veterinärplatz 1, 1210 Vienna, Austria; 2 Department of Veterinary Medical Imaging and Small Animal Orthopaedics, Ghent University, Ghent, Belgium; 3 Department of Radiology and Surgery, Faculty of Veterinary Medicine, University of Tehran, Tehran, Iran

**Keywords:** Head trauma, Computed tomography, Intravenous contrast administration, Dog

## Abstract

**Background:**

The aim of this study is to evaluate additional findings which can be detected by post-contrast computed tomography (CCT) in relation to plain CT (PCT) findings in patients presented with head trauma. Medical records of canine patients with the history of head trauma from three institutions were reviewed. PCT- and CCT-anonymized images were evaluated by a veterinary radiologist separately. From the categorized findings the following conclusions were drawn as: abnormalities were identified on (A) PCT but missed on CCT, (B) CCT but missed on PCT, (C) both PCT and CCT.

**Results:**

Thirty-two patients were included. The results showed that findings identified on CCT or PCT (category A and B) but missed on the other series were limited to mild soft tissue and sinus changes. Overall, 61 different fracture areas, 6 injuries of the temporomandibular joint (TMJ), 4 orbital injuries, 14 nasal cavities with soft tissue density filling, 13 areas of emphysema, 4 symphysis separations, 12 intracranial hemorrhages, 6 cerebral edema, 5 cerebral midline shifts, 3 intracranial aeroceles, 3 brain herniations and 6 intraparenchymal foreign bodies (defined as an abnormal structure located within the brain: e.g. bony fragments, bullet, teeth,..) were identified on both PCT and CCT separately (category C). Severity grading was different in 50% (3/6) of the reported cerebral edema using PCT and CCT images.

**Conclusion:**

The results showed that PCT is valuable to identify the presence of intracranial traumatic injuries and CCT is not always essential to evaluate vital traumatic changes.

## Background

Trauma is one of the most prevalent pathophysiologic processes affecting dogs [1]. Among patients referred to veterinary medical centers, high morbidity and mortality are reported in patients with traumatic head injuries [2, 3]. An immediate diagnostic evaluation is an important stage in this critical patient scenario. Among the diagnostic procedures, diagnostic imaging plays an important role in assessing the extent of head injury after clinical stabilization of the patient [3]. Computed tomography (CT) is the preferred modality for imaging in recent head trauma in comparison to the other modalities because it completely fulfills the need for a quick informative examination [4]. This preference of CT is due to its availability, the rapid examination, lower cost in comparison to magnetic resonance imaging (MRI), evaluation of the bones without superimposition, better visualization of preacute hemorrhage and evaluation of intracranial structures [2, 3]. Advance imaging offers a selective imaging approach to answer a specific clinical question. In this way, the selection of diagnostic imaging modality should be made based on the modality’s ability to demonstrate potential clinically important traumatic pathologies, its repeatability, costs and radiation exposure. As obtaining post contrast images require additional exposure to ionising radiation and have a small risk of reaction to the contrast medium, this study has been conducted to evaluate the additional findings that can be detected by post-contrast computed tomography (CCT) in addition to plain CT (PCT) findings in dogs presented with head trauma. We hypothesized that PCT would be sufficient to detect the major traumatic changes which may need intervention or management such as intracranial changes and fractures, and consequently demonstrate that CCT is not necessary in patients presenting with head trauma.

## Results

Thirty-two patients from 36 provided patients from the three centers were included in the study. In total 12 cases from diagnostic Imaging, clinic of small animals and horses, university of veterinary medicine (Vetmeduni), Vienna, Austria (2008–2018), 12 cases from the department of medical imaging and small animal orthopaedics, faculty of veterinary medicine, Ghent University, Ghent, Belgium (2010–2017) and 12 cases from department of radiology and surgery, faculty of veterinary medicine, University of Tehran, Tehran, Iran (2018–2019) were included considering the inclusion criteria. Four patients were excluded due to improper image qualities and motion artifacts.

**Fig. 1:**
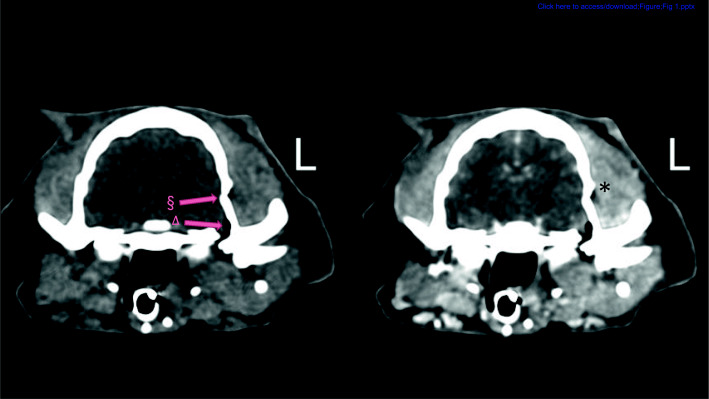
PCT (left) and CCT images (right) of a 14-year-old mixed-breed dog (Findings in category B). L: The patients’ left side. A mild soft tissue swelling (*) was scored only in PCT because of mild increased contrast uptake on the left side at the level of the calvarial fracture (§) in comparison with the other side. A small aerocele (∆) is. visible at the level of the fracture. WW (180) and WL (60) in these images are selected intermediately to show all the changes in one figure and avoid presenting several figures in the article

**Fig. 2:**
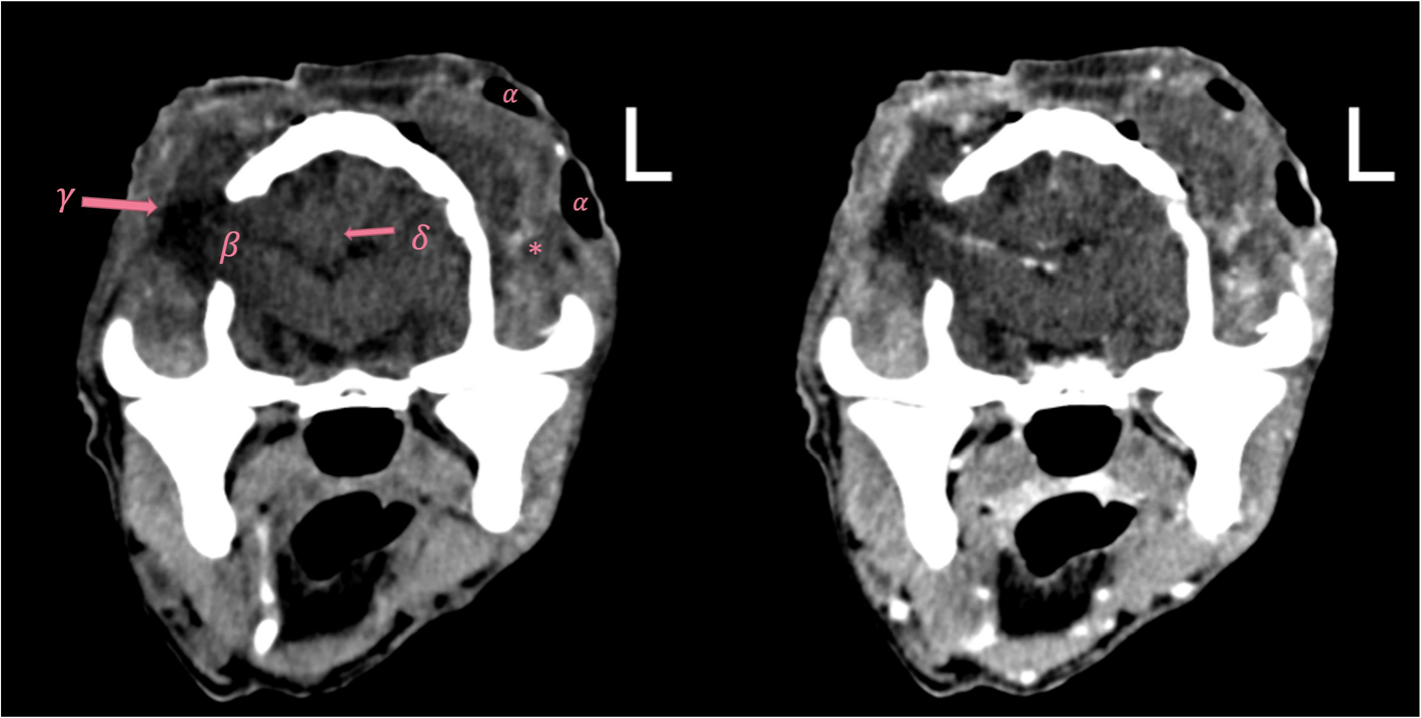
PCT (left) and CCT images (right) of a 10-year-old mixed-breed dog (Findings in category C). L: The patients’ left side. Emphysema (*α*), brain herniation (*γ*), skull fracture (*β*), cerebral midline shift (*δ*) and soft tissue changes (*), that are marked in the plain image (left), are detectable in both images. WW (180) and WL (60) in these images are selected intermediately to show all the changes in one figure and avoid presenting several figures in the article

**Fig. 3:**
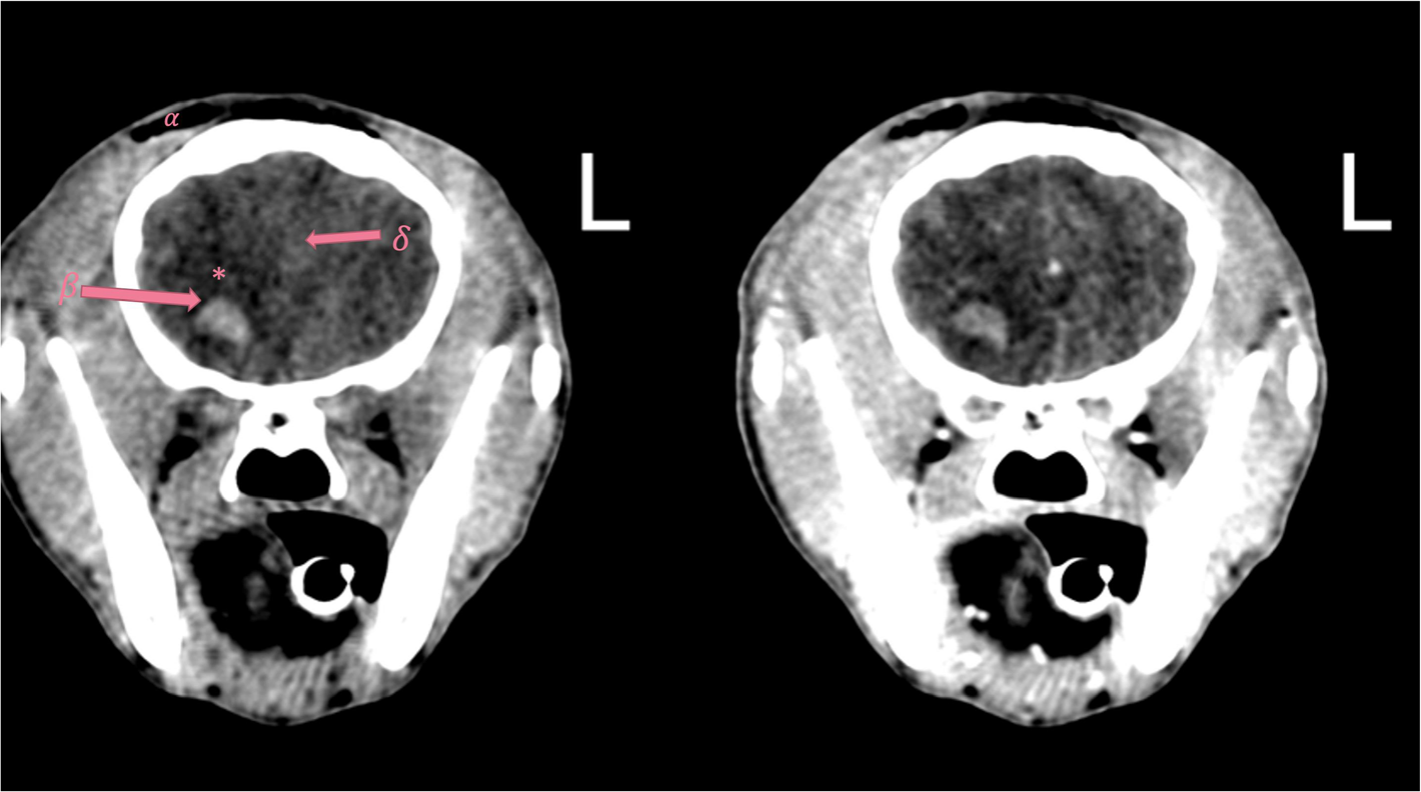
PCT (left) and CCT images (right) of a 5-month-old Jack Russel terrier (Findings in category C). L: The patients’ left side. Emphysema (*α*), intracranial hemorrhage (*β*), cerebral edema (*), and mild cerebral midline shift (*δ*), that are marked in the plain image (left), are detectable in both images. WW (180) and WL (60) in these images are selected intermediately to show all the changes in one figure and avoid presenting several figures in the article

Dogs had a mean age of 47.7 months at time of the presentation (median: 36 months, range: 2 to 132 months), ﻿22 dogs were male, 8 dogs were female, and the gender was not recorded in 2 dogs. Breeds included mixed breed (*n* = 10), Chihuahua (*n* = 4), Shih Tzu (*n* = 4), German Shepherd (*n* = 3), Boomer (*n* = 1), Spitz (*n* = 1), Vizsla (*n* = 1), Yorkshire terrier (*n* = 1), Boxer (*n* = 1), Jack Russell terrier (*n* = 1), Lhasa Apso (*n* = 1), Weimaraner (*n* = 1), Doberman pinscher (*n* = 1), Border terrier (*n* = 1) and Pomeranian (*n* = 1). Overall 166 lesions were detected. The results showed that findings identified on CCT or PCT but missed on the other series were limited to mild soft tissue and sinus changes (category A and B) (Table 1, Fig. 1). Overall, 61 different fractures, 6 temporomandibular joint (TMJ) injuries including fractures and luxations, 4 orbital injuries (exophthalmos and globe deterioration), 14 nasal cavities with soft tissue density filling, 13 emphysema, 4 symphysis separations, 12 intracranial hemorrhages, 6 cerebral edema, 5 cerebral midline shift, 3 intracranial aerocels, 3 brain herniations and 6 intraparenchymal foreign bodies (defined as an abnormal structure located within the brain e.g. bony fragments, bullets, teeth, ….) were identified on both PCT and CCT separately (category C, Figs. 2 and 3) (Tables 1, 2 and 3). The Cohen’s Kappa test showed an almost perfect agreement, κ= 1 (95% CI, *p* < .0001), between PCT and CCT in detection of fractures, TMJ injuries, orbital injuries, nasal cavity filling, emphysema, symphysis separation, intracranial hemorrhage, cerebral edema, cerebral midline shift, intraparenchymal aerocele, brain herniation and intraparenchymal foreign bodies. The Cohen’s Kappa test revealed less agreement between PCT and CCT in detection of soft tissue and sinus involvements in comparison to the other lesions. The Kappa value showed a moderate and strong agreement between PCT and CCT in detection of soft tissue and sinus involvements respectively, κ= 0.78 and 0.81 respectively (95% CI, *p* < .0001).

**Table 1:** PCT and CCT assessment of head injury: extracranial lesions

	Soft tissue involvement	Sinus involvement	Nasal cavity filling	Emphysema	Orbital injury
(A) PCT negative, CCT positive	2	1	0	0	0
(B) CCT negative, PCT positive	1	1	0	0	0
(C) CCT and PCT positive	18	6	14	13	4
Total	21	8	14	13	4

**Table 2:** PCT and CCT assessment of head injury: intracranial lesions

	Intracranial hemorrhage	Cerebral edema	Midline Shift	Intraparenchymal foreign body	Aerocele	Brain herniation
(A) PCT negative, CCT positive	0	0	0	0	0	0
(B) CCT negative, PCT positive	0	0	0	0	0	0
(C) CCT and PCT positive	12	6	5	6	3	3
Total	12	6	5	6	3	3

**Table 3:** PCT and CCT assessment of head injury: bone changes

	Fractures	TMJ injury	Symphyseal injury
(A) PCT negative, CCT positive	0	0	0
(B) CCT negative, PCT positive	0	0	0
(C) CCT and PCT positive	61	6	4
Total	61	6	4

The severity of the reported cerebral edema was graded differently using PCT and CCT in 50% (3/6) of the patients, and in all these patients the edema severity was graded more in CCT.

## Discussion

The main result of the evaluation of traumatic changes in plain and contrast CT-images of 32 dogs with a history of head trauma showed that mild soft tissue and sinus involvements are the only findings which may be detected only in one of the plain or post-contrast series. The agreement between PCT and CCT is lower in detection of soft tissue and sinus involvements, however there is still a moderate and strong agreement respectively.

Recently, advances in diagnostic imaging technology and knowledge offers new approaches in diagnosis and patient management both in human medicine and veterinary medicine. A challenge of “cost-effective” and “health-benefit” is always an ongoing discussion in any case management to select proper diagnostic tests [5]. This challenge becomes more complicated when it is combined with time limitations in the case of emergencies and trauma [6, 7]. Following the utilization of plain CT without contrast administration in human medicine to assess traumatic changes in the head [8], some veterinary radiologists also prefer to skip CT angiography in head trauma patients based on anecdotal evidence [6]. Therefore, this study was conducted to evaluate extra information obtained by post-contrast computed tomography (CCT) in relation to plain CT (PCT) findings and to determine the necessity of contrast medium administration in patients presented with head trauma.

CT is already known as an valuable diagnostic tool for cases of acute head trauma because it shows small bony changes and intracranial hemorrhage [4]. In the present study plain CT could detect the intracranial lesions and bony changes (fractures, TMJ injuries and symphysis separations), and none of the detected lesions were exclusively visible on post-contrast images. Therefore, based on the present findings, PCT alone is felt to be an informative to evaluate the major traumatic changes.

Traumatic brain injury (TBI) is a life-threatening consequence of head trauma and can result in death and is responsible for high mortality (18 to 24%) in dogs with trauma [9, 10]. Typically, death in TBI results from progressive increase in intracranial pressure (ICP) [3, 11, 12]. Recently, a noninvasive imaging-based method such as CT is suggested for ICP monitoring or screening tool for patients with concern of severe TBI in human medicine [13]. Some gross anatomic changes such as intracranial occupying masses or hematomas, enlarged ventricles, cerebral edema, ventricular compression and midline shift are recognised in human medicine associated with elevated ICP [14]. To the best of the authors’ knowledge, this method is not validated in veterinary medicine. However, it is logical to consider that the same secondary changes in the brain can be present as indicators of increased ICP in dogs as well. All these gross anatomical changes were detected in the present plain image series as well as in the post-contrast images. Thus, the plain CT can help the clinicians to check if secondary changes are seen that may be associated to the increased ICP.

Cerebral edema, including vasogenic edema and cytotoxic edema, results in an increase of brain volume and hypoattenuating changes of the parenchyma [11, 15]. The incidence of cerebral edema, brain hernia and midline shift as secondary signs of increased brain volume were detected in the present cases on plain CT. Based on our experience the plain images are still diagnostic, however, these changes are more marked in the post-contrast images, which were more obvious in brain edema scoring. No gold standard was present in this study to proof the presence of the subtle cerebral edema which is detectable in CT. The authors’ propose that if edema is not detectable on pre-contrast CT, then it is not detectable in post-contrast images neither.

The mild soft tissue and sinus involvements are the only radiologic findings which were categorized in categories (A) and (B). These findings were detected in PCT but missed on CCT or were detected in CCT but missed on PCT. As the patients in this study were randomly included, it is not clear whether the distribution of all scores of soft tissue and sinus involvements was equal and normal or not. So, it is admitted that the effect of the extension and severity of the sinus involvement, soft tissue swelling on these results is unclear. In the case of the clinically relevance and importance of these injuries, agreement of PCT and CCT should be assessed in light of the presenting clinical signs and patient outcome.

Due to the retrospective study design this study has limitations. The “Modified Glasgow Coma Scale (MGCS)” is a prognostic indicator and monitoring tool in the patients with head trauma in veterinary medicine [16]. MGCS was not included as the patients were collected from different hospitals and some of the patients were referrals and MGCS was not mentioned in medical records. As the aim of this study was not the utilization of plain and contrast CT images as a prognostic tool, the results do not lead to a final judgment concerning the necessity of the contrast administration as a prognostic tool. Because of the retrospective and multicentral design of the study, different acquisition settings, different reconstructions of the raw data were used that may have influenced the image quality. The study aim was to compare pre- and post-contrast findings in each patient, thus differences in image acquisition were not a concern for the present study. Furthermore, an inhomogeneous sample group (patients with different breed, size and age) and different source of trauma could be considered as a potential uncontrolled limitation, however the authors believe it does not affect the main aim and results of this study.

Additionally, the scoring was subjective in the present study and no gold standard (e.g. necropsy or histopathology) was used to proof the scoring. The better enhancement of the soft tissue and the effect of windowing the images in CCT should be considered as a bias and a possible reason of detecting mild soft tissue and sinus involvements only in one, PCT or CCT.

## Conclusion

In conclusion, the results showed that PCT is valuable to identify the presence of intracranial traumatic injuries and CCT is not always essential to evaluate vital traumatic changes. In cases which soft tissue involvement or sinus involvement are expected, evaluation of both PCT and CCT images is advised. The results may be helpful in dealing with the challenge of planning of the investigations, while considering the side effects, longer anesthesia and extra price for CT-angiography in the evaluation of the skull trauma. Evaluation of the extension and clinical importance of detected lesions are recommended in further investigations.

## Methods

The study was a retrospective, multicenter and descriptive design. Due to the retrospective study design, no institutional animal care and use approvals were requested officially. Dogs were included if the medical files showed a history of trauma as an indication for CT scans. Trauma was defined as any tissue injury that occurred suddenly as a result of an external force, including blunt force injury (road traffic accident, fall from a height or kicked by horse), penetrating injury (gunshot and another animal incident or bite), or crushing injury. Cases with unknown or questionable history of trauma or concurrent neoplasia in skull were excluded. Cases were included if nose to 1st cervical vertebrae were included in the image series and both plain and post-contrast image series were available for the evaluation. Studies which had inadequate image quality were excluded.

Three Institutions: 1. diagnostic Imaging, clinic of small animals and horses, university of veterinary medicine (Vetmeduni), Vienna, Austria using a SOMATOM®Emotion-16 detectors CT scanner (Siemens Healthcare, Erlangen, Germany); 2. department of medical imaging and small animal orthopaedics, faculty of veterinary medicine, Ghent University, Ghent, Belgium using a helical CT scanner (LightSpeed, GE Medical Systems, Milwaukee, WI); 3. department of radiology and surgery, faculty of veterinary medicine, University of Tehran, Tehran, Iran using a SiemensSomatom®- two detectors CT scanner (Siemens Healthcar, Erlangen, Germany); were participated in the present study. The images were taken with different scan parameters and contrast medium administrations.

The archives of all these institutions were investigated retrospectively for all dogs which underwent CT of the head.

Plain and contrast image series of the included cases were retrieved and anonymized separately. The image series were reviewed by one radiologist (YV) in random order within 3 months and pre- and postcontrast studies for each patient reviewed blindly on separate occasions. All the images were reviewed with the assessed window width (WW) and window level (WL) depending on the evaluated structures, using a Miele-LXIV DICOM Workstation and Image Viewer (version 7.5.52, Alex Bettarini (bettar)).

The traumatic changes were defined in three main categories: (1) intracranial lesions, (2) extracranial lesions, and (3) bony changes. Furthermore, these three categories were subjectively subcategorized and scored based on severity (non, mild, moderate and severe) and location (Table 4). Comparison of the results of PCT vs CCT for each subcategory was made by matching the image series and scores. Finally, the following conclusions were drawn from direct comparisons of the results of PCT and CCT from each individual dog: abnormalities were identified on (A) PCT but missed on CCT, (B) CCT but missed on PCT, (C) both PCT and CCT (Tables 1, 2, 3).

**Table 4:** Categories, subcategories, recording and scoring guideline used in evaluation of the traumatic findings in the present study

Bony changes	Fracture	Region	Bones of the brain case	Ethmoid	1
				Frontal	2
				Occipital	3
				Parietal	4
				Sphenoid	5
				Temporal	6
			Bones of the face and palate	Lacrimal	7
				Mandible	8
				Maxilla	9
				Nasal	10
				Palatine	11
				Pterygoid	12
				Vomer	13
				Zygomatic	14
		Type	Compressed	No	0
				Yes	1
			Non-Compressed	No	0
				Yes	1
	TMJ		Type	Normal	0
				Subluxation	1
				Luxation	2
				Fracture	3
	Symphyseal Injury			No	0
				Yes	1
Intraacranial lesions	Intracranial hemorrhage		At the level of bone involvement	No	0
				Yes	1
			Area without bone involvemengt	No	0
				Yes	1
	Cerebral midline shift		Location	Right	1
				Left	2
			Severity	No	0
				Mild	1
				Moderate	2
				Severe	3
	Cerebral edema		Location	Right	1
				Left	2
			Severity	No	0
				Mild	1
				Moderate	2
				Severe	3
	Intraparenchymal foreign body			No	0
				Yes	1
	Aerocele			No	0
				Yes	1
	Brain herniation			No	0
				Yes	1
Extracranial lesions	Orbital involvement		Exophtalmus	No	0
				Unilateral	1
				Bilateral	2
			Globe deterioration	No	0
				Unilateral	1
				Bilateral	2
	Soft tissue swelling		Severity	No	0
				Mild	1
				Moderate	2
				Severe	3
	Emphysema		Severity	No	0
				Mild	1
				Moderate	2
				Severe	3
	Frontal sinus content		Severity	No	0
				Less than 30% (Mild)	1
				30–60% (Moderate)	2
				30–100% (Severe)	3
			Sides	Unilateral	1
				Bilateral	2
	Nasal content		Severity	No	0
				Less than 30% (Mild)	1
				30–60% (Moderate)	2
				30–100% (Severe)	3
			Sides	Unilateral	1
				Bilateral	2

The agreement between PCT and CCT in identification of the traumatic changes were evaluated by Cohen’s kappa test using SPSS (version 19.0; IBM, Chicago, USA). The result of the Cohen’s Kappa test were interpreted based on the guideline presented by McHugh 2012 [17].

## Data Availability

The datasets used and/or analysed during the current study available from the corresponding author on reasonable request.

## Abbreviations

CCT: Post-contrast computed tomography
CT: Computed tomography
ICP: Intracranial pressure
MGCS: Modified glasgow coma scale
PCT: Pre-contrast (plain) computed tomography
TBI: Traumatic brain injury
TMJ: Temporomandibular joint
WL: Window level
WW: Window width

## References

[1] Dan GO, Church DB, McGreevy PD, Thomson PC, Brodbelt DC (2014). Prevalence of disorders recorded in dogs attending primary-care veterinary practices in England. PLoS One.

[2] Hall K (2011). Canine trauma: literature review and evidence based medicine. J Vet Emerg Crit Care.

[3] Dewey CW (2000). Emergency management of the head trauma patient: principles and practice. Vet Clin.

[4] Dennis R (2003). Advanced imaging: indications for CT and MRI in veterinary patients. In Practice.

[5] Elstein AS, Schwartz A (2002). Clinical problem solving and diagnostic decision making: selective review of the cognitive literature. Br Med J.

[6] Schwarz T. Is speed everything? Use of CT for the emergency patient. In: BSAVA Congress Proceedings 2017. Birmingham: BSAVA Library; 2017. p. 58–9.

[7] Bruce DA (2000). Imaging after head trauma: why, when and which. Childs Nerv Syst.

[8] Naraghi L, Larentzakis A, Chang Y, Duhaime AC, Kaafarani H, Yeh DD, King DR, de Moya MA, Velmahos GC (2015). Is CT angiography of the head useful in the management of traumatic brain injury?. J Am Coll Surg.

[9] Sharma D, Holowaychuk MK (2015). Retrospective evaluation of prognostic indicators in dogs with head trauma: 72 cases (January–march 2011). J Vet Emerg Crit Care.

[10] Simpson SA, Syring R, Otto CM (2009). Severe blunt trauma in dogs: 235 cases (1997–2003). J Vet Emerg Crit Care.

[11] DiFazio J, Fletcher DJ (2013). Updates in the management of the small animal patient with neurologic trauma. Vet Clin.

[12] Sande A, West C (2010). Traumatic brain injury: a review of pathophysiology and management. J Vet Emerg Crit Care.

[13] Pappu S, Lerma J, Khraishi T (2016). Brain CT to assess intracranial pressure in patients with traumatic brain injury. J Neuroimaging.

[14] Harary M, Dolmans RG, Gormley WB (2018). Intracranial pressure monitoring -review and avenues for development. Sensors..

[15] Schwarz T, Saunders J, editors. Calvarium and zygomatic arch. In: Veterinary computed tomography. West Sussex: Wiley; 2011.

[16] Elias N, Rotariu AM, Grave T (2019). Traumatic brain injury in dogs and cats. Companion Anim.

[17] McHugh ML (2012). Interrater reliability: the kappa statistic. Biochem Med.

[18] Vali Y, Gielen I, Soroori S, Ludewig E. The necessity of intravenous contrast CT in addition to plain CT in dogs with head trauma. Abstracts of the European Veterinary Diagnostic Imaging (Evdi) Congress, Basel, Switzerland, August 21–August 24, 2019. Vet Radiol Ultrasound. 2019;61(1):1–34.

